# A new species of *Hypoaspis* Canestrini (Acari, Mesostigmata, Laelapidae) associated with *Oryctes* sp. (Coleoptera, Scarabaeidae) in Iran

**DOI:** 10.3897/zookeys.574.7767

**Published:** 2016-03-28

**Authors:** Omid Joharchi, Alireza Shahedi

**Affiliations:** 1 Young Researchers and Elite Club, Yazd Branch, Islamic Azad University, Yazd, Iran; 2 Department of Plant Protection, College of Agriculture, Shiraz University, Shiraz, Iran

**Keywords:** Gamasina, Dermanyssoidea, taxonomy, *Hypoaspis surenai* sp. n., Hypoaspidinae, Taft, Yazd

## Abstract

A new species of the genus *Hypoaspis* Canestrini, *Hypoaspis surenai* **sp. n.**, is described based on adult female specimens collected in association with *Oryctes* sp. (Coleoptera: Scarabaeidae) in Taft, Yazd province, Iran.

## Introduction

The mite family Laelapidae includes approximately 800 species of morphologically, ecologically and behaviourally very diverse dermanyssoid mites, including obligate and facultative parasites of vertebrates, insect paraphages, and free-living predators that inhabit soil-litter habitats and the nests of vertebrates and arthropods (Evans and Till 1966; Faraji and Halliday 2009; Lindquist et al. 2009; Joharchi et al. 2011; Joharchi et al. 2012a, b). Currently, the family is classified into approximately 144 genera, including *Hypoaspis* with 36 species.

Joharchi and Halliday (2011) treated *Hypoaspis* *sensu stricto* as a separate genus equivalent to Hypoaspis (Hypoaspis) of other authors (e.g., Evans and Till 1966; Karg 1979, 1982, 1993), and gave a diagnosis and comparison of diagnostic characters for the closely related genus *Coleolaelaps* Berlese. That concept of *Hypoaspis* *s.s.* is followed here. The most recent taxonomic work on the genus was by Joharchi et al. (2014), who clarified the diagnosis of the genus and reviewed species that occur in the Western Palaearctic Region. In Iran, *Hypoaspis* *s.s.* included 14 identified species prior to this study (Joharchi and Halliday 2011; Razavi Susan et al. 2014; Joharchi et al. 2014).

The ecological role of this genus is unknown. They may feed on exudates from the beetle’s body or their eggs, or on other small invertebrates in the microhabitats created by the beetles (Costa 1971; Joharchi and Halliday 2011; Joharchi et al. 2014). This has not been established experimentally, and it will be necessary to do feeding experiments to establish the true ecological role of these mites. The purpose of this paper is to describe another species of *Hypoaspis* *s.s.* to increase our knowledge of the Iranian fauna of Laelapidae.

## Materials and methods

Phoretic laelapids on beetles were collected from Taft, Yazd province, Iran, in 2015. Mites were removed from the beetles using an entomological pin. Specimens were cleared in Nesbitt’s solution and mounted in Hoyer’s medium (Walter and Krantz 2009). The line drawings and examination of the specimens were performed with an Olympus BX51 phase contrast microscope equipped with a drawing tube and figures were elaborated with Corel X-draw software, based on the scanned line drawings. Dorsal shield length and width were taken from the anterior to posterior margins along the midline, and at its broadest point, respectively. Length and width of the sternal shield were measured from the anterior border to the posterior margin at the full length and broadest point, respectively. Genital shield length and width were measured along the midline from the anterior border of the genital shield to the posterior margin of the genital shield, and at the maximum, respectively. Leg lengths were measured from base of the coxa to the apex of the tarsus, excluding the pre-tarsus. The nomenclature used for the dorsal idiosomal chaetotaxy is that of Lindquist and Evans (1965), the leg chaetotaxy is that of Evans (1963a), the palp chaetotaxy is that of Evans (1963b), and names of other anatomical structures mostly follow Evans and Till (1979). We use the terms “lyrifissures” to refer to slit-shaped sensilli, “gland pores” to refer to structures that we believe are the openings of secretory pores, and “poroids” for circular or oval-shaped cuticular openings of unknown function. The holotype (ARS-20150304-1a) and six paratypes (ARS-20150304-1b, ARS-20150304-1c, ARS-20150304-1d, ARS-20150304-1e, ARS-20150304-1f, ARS-20150304-1g) of the new species are deposited in the Acarological Collection, Department of Plant Protection, Yazd Branch, Islamic Azad University (YIAU). Two paratypes (ARS-20150304-1h, ARS-20150304-1i) are deposited in the Jalal Afshar Zoological Museum, College of Agriculture, University of Tehran, Iran (JAZM) and two paratypes (ARS-20150304-1k, ARS-20150304-1l) are also in the Australian National Insect Collection, CSIRO, Canberra, Australia (ANIC). All measurements in the descriptions are given in micrometres (μm).

## Taxonomy

### Genus Hypoaspis

Taxon classificationAnimaliaColeopteraScarabaeidae

Canestrini

Hypoaspis Canestrini, 1884: 1569.

#### Type species.

*Gamasus krameri* G. & R. Canestrini, 1881, designated by Berlese (1904).

The short diagnosis below is summarised from the detailed diagnosis in Joharchi and Halliday (2011).

#### Short diagnosis.

Dorsal shield oval, without lateral incisions, bearing 35–40 pairs of setae including one or more pairs of *Zx* setae; some opisthonotal setae greatly elongated, especially *Z4* (at least three times as long as *J4*); post-anal seta distinctly shorter than para-anals; hypostomal setae *h3* distinctly longer than other hypostomal setae; tarsus II with two subterminal blunt spines (setae *al1* and *pl1*).

### Hypoaspis surenai sp. n.

Taxon classificationAnimaliaColeopteraScarabaeidae

http://zoobank.org/11977B75-8434-4596-A481-782332BE2541

Figures 1–7

#### Type material.

Holotype, female, **Iran**, Yazd Province, Taft, Kahduiyeh, 31°16'N, 53°43'E, alt. 1496 m a.s.l, 04March 2015, A. Shahedi coll., on adult females of *Oryctes* sp. (Coleoptera: Scarabaeidae). Paratypes: ten females same data as holotype.

#### Description of the female.

**Figures 1–7.**
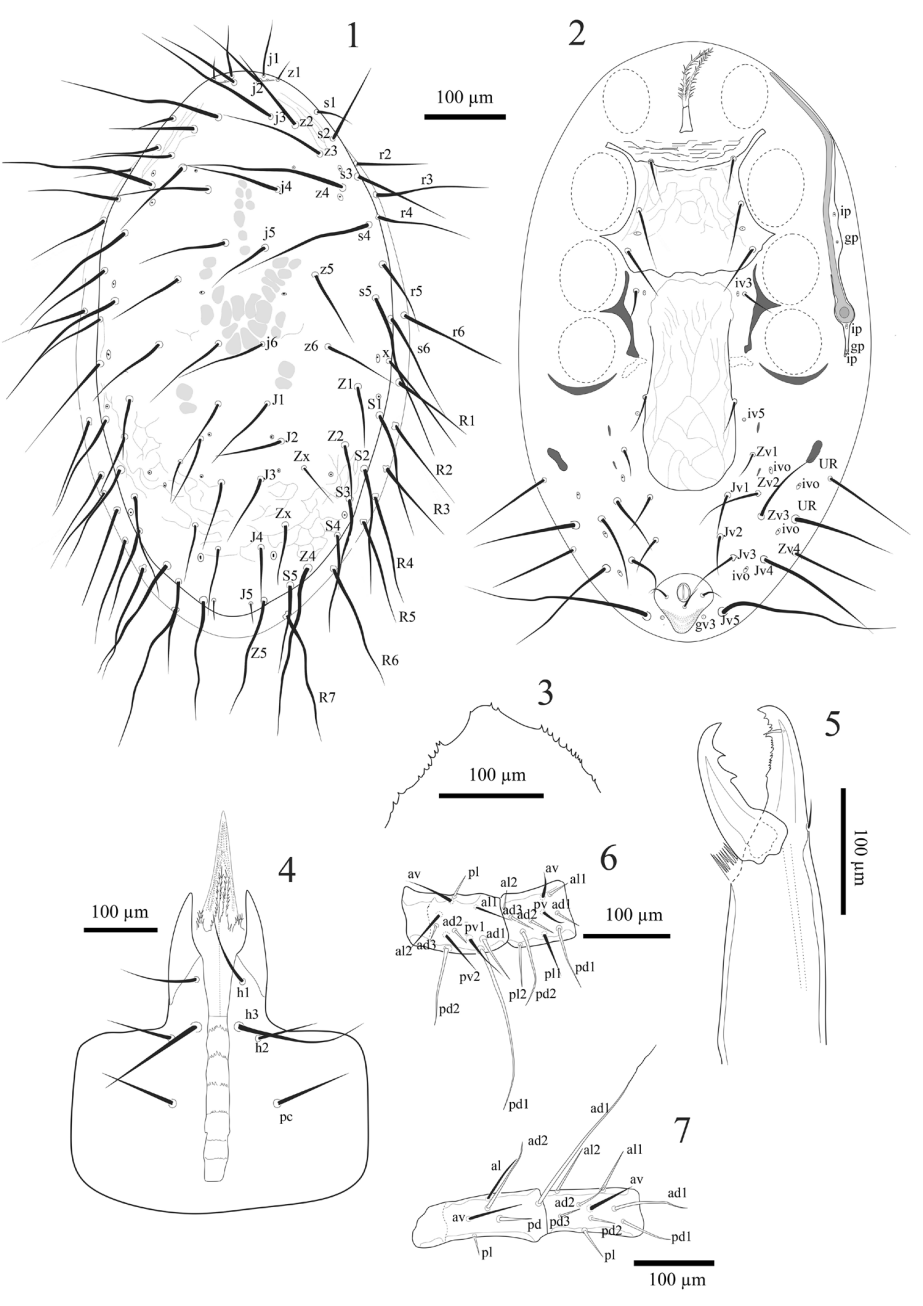
*Hypoaspis surenai* sp. n., female **1** dorsal idiosoma **2** ventral idiosoma **3** epistome **4** Subcapitulum **5** chelicera **6** femur and genu II **7** femur and genu IV.

*Dorsal idiosoma* (Fig. 1). Length 796–802, width at level of *r5*, 446–450. Dorsal shield oval, without lateral incisions, length 778–785, width at level of *r5*, 420–426 (n= 11), shield without distinct reticulate ornamentation over whole surface, only with weak reticulation, more distinct in opisthonotal region (Fig. 1). Dorsal shield with 37 pairs of smooth and pointed setae, 21 pairs on podonotal shield (*j1–6*; *z1–6*; *s1–6*; *r4–5* and including a supernumerary pair near *s6*), plus *r2*, *r3* and *r6* off the shield in the soft skin, 16 pairs on the opisthonotal shield (*J1–5*, *Z1*, *Z2*, Z4, Z5, *S1–5*), including two pairs of *Zx* setae between *J* and *Z* setae, seta *Z3* absent (Fig. 1); *Z4* longest (322–330) and slightly wavy, *s5* (219–225), *s4* (198–207), *z4* (232–245) and *j3* (222–230) also long, *j1* (74–75) and *z1* (30–31) short; *j4* (128–132) long enough to reach past *j5*, *j5* (112–117) not long enough to reach *j6*, *j6* (138–142) not long enough to reach *J2* (100–108) but reaching past *J1* (118–123); *J4* (98–100) long enough to reach *J5* (27–29); *Z5* (178–180) and *S5* (136–139) also long. Seven pairs of setae in *R* series on the lateral area of weakly sclerotised cuticle surrounding shield; *R7* elongate (182–191) and appearing wavy. Shield with 12 pairs of pore-like structures, apparently including four pairs of gland pores and eight pairs of poroids; lyrifissures near the base of *j1* large and slit-like, others smaller and ovoid.

*Ventral idiosoma* (Fig. 2). Tritosternum with paired pilose laciniae (141–143), columnar base 30–32 long, 20–21 wide; pre-sternal area weakly reticulated. Sternal shield (length 138–148) narrowest between coxae II (138–148), widest between coxae II–III (198–200), with slightly concave anterior margin and irregular posterior margin, with three pairs of long, smooth pointed setae (*st1* 52–54, *st2* 82–84, *st3* 74–79), *st2* and *st3* reaching well past base of next posterior setae, and two pairs of lyrifissures, one pair adjacent to *st1*, the other between *st2* and *st3*, lateral and central surface of sternal shield with weak reticulation. Metasternal platelets absent, metasternal setae *st4* (45–47) and metasternal poroids located on weakly sclerotised cuticle. Endopodal plates II/III completely fused to sternal shield, endopodal plates III/IV roughly triangular and curved. Genital shield tongue-shaped, length 278–280, maximum width 118–120, posterior margin rounded, surface with reticulate ornamentation, genital setae *st5* (50–52) on edge of the shield. Circular paragenital poroids located on weakly sclerotised cuticle close to *st5*. Anal shield rounded triangular, length 87–89, width 87–89, para-anal (39–41) and post-anal (38–40) setae equal in length, cribrum small, a pair of circular lateral gland pores flank anal shield. Opisthogaster with one pair of oval metapodal plates (22–23 long × 5–7 wide) and 11 pairs of smooth setae on the weakly sclerotised cuticle; *Jv1*, *Jv2* 70–72, *Jv3* 89–92, *Jv4* 158–160, *Zv1* 50–52, *Zv2* 91–93, *Zv3*, *Zv4* 100–105, *UR* 124–126, *Jv5* 242–250 very long and wavy. Exopodal plates behind coxa IV long and narrow. Peritrematal shield free posteriorly, with large protrusion on outer margin opposite coxae II–III bearing two pairs of pore-like structures (apparently one lyrifissure ‘*ip*’, and one gland pore ‘*gp*’; see Fig. 2), post-stigmatal section conspicuous and narrow, with three pairs of pore-like structures of post-stigmatal pores (apparently two lyrifissures ‘*ip*’, and one gland pore ‘*gp*’; see Fig. 2), peritreme extending from posterior margin of coxa III to near mid level of coxa I.

*Gnathosoma*. Epistome irregularly denticulate laterally, apical section smooth with minute denticles in some specimens (Fig. 3). Hypostomal groove with six rows of 6–11 denticles, and smooth anterior and posterior transverse lines. Hypostome with four pairs of setae, internal posterior hypostomal setae *h3* longest (109–110), *h1* (54–55), *h2* (45–47), palpcoxal *pc* (52–54) (Fig. 4). Corniculi robust and horn-like, reaching mid-level of palp femur. Palp setal numbers: trochanter 2, femur 5, genu 6, tibia 12, tarsus 15, all setae smooth and pointed, palp tarsal apotele two-tined. Internal malae complex, with two pairs of lobes, inner lobes narrow, with serrated edges, outer lobes narrow, pointed, shorter than inner lobes, with serrated edges (Fig. 4). Fixed digit of chelicera with 15 small teeth, the one level with the pilus dentilis largest (Fig. 5), pilus dentilis short and robust, dorsal seta short, semi-erect, movable digit with two large subterminal teeth, arthrodial membrane a rounded flap with a corona and cheliceral lyrifissure indistinct.

*Legs*. Legs II and III shortest (564–570, 604–610), I and IV both longer (702–711, 872–880) (excluding pretarsus). Chaetotaxy normal for free-living Laelapidae. Leg I: coxa 0-0/1, 0/1-0, trochanter 1-0/1, 1/2-1, femur 2-3/1, 2/3-2, genu 2-3/2, 3/1-2, tibia 2-3/2, 3/1-2. Leg II: coxa 0-0/1, 0/1-0, trochanter 1-0/1, 0/2-1, femur 2-3/1, 2/2-1 (macrosetae *pd1* 184–190, *pd2* 94–97, Fig. 6), genu 2-3/1, 2/1-2 (*pd1* 84–86 and *pd2* 100–103 longer, Fig. 6), tibia 2-2/1, 2/1-2. Leg III: coxa 0-0/1, 0/1-0, trochanter 1-1/1, 1/1-0, femur 1-2/1, 1/0-1 (macroseta *ad1* 124–128; *ad2* longer 37–43), genu 2-2/1, 2/1-1 (*ad1* 57–59 and *pd1* 90–94 longer), tibia 2-1/1, 2/1-1 (ventral setae all thicker). Leg IV: coxa 0-0/1, 0/0-0, trochanter 1-1/1, 0/1-1, femur 1-2/1, 1/0-1 (macroseta *ad1* 200–207, *ad2* longer 90–92, Fig. 7), genu 2-2/1, 3/0-1 (*ad1* 84–86 and *pd1* 60–62 longer, Fig. 7), tibia 2-2/1, 3/1-2. Tarsi II–IV with 18 setae 3-3/2, 3/2-3 + *mv*, *md*. On tarsus II, *al1*, *pl1* and all ventral setae thicker. Tarsus IV with three macrosetae, *ad2* (164–169), *pd2* (100–107) and *pd3* (142–147) and *pl3* thick. All pre-tarsi with a pair of claws and a long thin membranous ambulacral stalk.

*Genital structures*. Insemination ducts opening on posterior margin of coxa III, sacculus indistinct, apparently unsclerotised.

#### Males & immature.

Unknown.

#### Etymology.

The species is named in memory of Surena (died 53 BC) was a Parthian spahbed (“General” or “Commander”) during the 1^st^ century BC.

#### Remarks.

According to the key to species of *Hypoaspis* *s.s.* occurring in the Western Palaearctic Region provided by Joharchi et al. (2014), *Hypoaspis surenai* most resembles *Hypoaspis pentodoni* Costa, 1971 but has the following unique character states for the genus: 21 pairs of long smooth, pointed setae on the podonotal shield, including a supernumerary pair near *s6* (x) and *r2*, *r3*, *r6* off the shield; 16 pairs of smooth and long setae on the opisthonotal shield including two pairs of *Zx* setae between the *J* and *Z* setae, seta *Z3* absent; three long macrosetae on tarsus IV (*ad2*, *pd2* and *pd3*); one macroseta on each of femora II–IV and seta *ad1* on genu IV being only slightly longer than the remaining setae on the segment.

## Discussion

Fifteen species regarded to belong to *Hypoaspis* *s.s.* had been reported from Iran until now (including the new species): *Hypoaspis alborzensis* Razavi Susan & Joharchi, 2014; *Hypoaspis campestris* (Berlese, 1887) *sensu* Bregetova, 1977; *Hypoaspis elegans* Joharchi et al. 2014; *Hypoaspis integer* Berlese, 1911; *Hypoaspis krameri* (G. & R. Canestrini, 1881); *Hypoaspis larvicolus* Joharchi & Halliday, 2011; *Hypoaspis maryamae* Joharchi & Halliday, 2011; *Hypoaspis melolonthae* Joharchi & Halliday, 2011; *Hypoaspis neokrameri* Costa, 1971; *Hypoaspis pentodoni* Costa, 1971; *Hypoaspis phyllognathi* Costa, 1971; *Hypoaspis polyphyllae* Khanjani & Ueckermann, 2005; *Hypoaspis rhinocerotis* Oudemans, 1925; *Hypoaspis surenai* sp. n.; *Hypoaspis terrestris* (Leonardi, 1899).

Almost all of the species of *Hypoaspis* *s.s.* occurring in Iran are associated with Coleoptera, especially with a wide variety of species in the family Scarabaeidae, while a few have been collected in soil. Most of these species have been collected on only a few occasions, so it is difficult to draw any firm conclusions about their host specificity. The question of host or microhabitat specificity of the species cannot be analysed in detail until all of the available collections are re-examined to confirm the identifications.

## Supplementary Material

XML Treatment for Hypoaspis

XML Treatment for Hypoaspis surenai
